# Perceiving More Age-Related Losses? The Role of General Control Beliefs and Daily Disturbance to Plans

**DOI:** 10.1093/geroni/igab046.1107

**Published:** 2021-12-17

**Authors:** Shevaun Neupert, Xianghe Zhu

**Affiliations:** 1 North Carolina State University, Raleigh, North Carolina, United States; 2 North Carolina State University, North Carolina State University, North Carolina, United States

## Abstract

We examined daily fluctuations in future time perspective within the daily stress and awareness of aging processes. Awareness of age-related change (AARC) focuses on everyday experiences that highlight changes in behavior and functioning as a result of growing older. We integrated individual differences in control beliefs because those with higher control tend to be more resilient to stressors. We conducted a daily diary study of 112 older adults (aged 60-90) who completed measures of control beliefs at baseline and then daily measures of stressor exposure, appraisal (e.g., threats to future plans), and AARC for eight consecutive days. Increases in threats to future plans were associated with increases in AARC losses, and those with low control were especially vulnerable to increases in threats to future plans. With a constricted future time perspective, any threats to future plans may be especially harmful for older adults who are low in control beliefs.

